# tRNA‐derived fragments are promising biomarkers for screening of early colorectal cancer

**DOI:** 10.1002/mco2.227

**Published:** 2023-05-03

**Authors:** Yanqi Dang, Liang Dai, Jingjuan Xu, Wenjun Zhou, Yangxian Xu, Guang Ji

**Affiliations:** ^1^ Institute of Digestive Diseases Longhua Hospital China‐Canada Center of Research for Digestive Diseases (ccCRDD) Shanghai University of Traditional Chinese Medicine Shanghai China; ^2^ Clinical Research Academy Peking University Shenzhen Hospital Peking University Shenzhen China; ^3^ Department of General Surgery Longhua Hospital Shanghai University of Traditional Chinese Medicine Shanghai China

Dear Editor,

Colorectal cancer (CRC) is the third most common cancer and the second most common cause of cancer‐related death globally.^1^ In the clinical setting, early screening of CRC is vital for subsequent management. Conventional serum biomarkers such as carcinoembryonic antigen (CEA) and carbohydrate antigen 19‐9 (CA19‐9) are widely employed, but their diagnostic performance for early‐stage CRC is not satisfactory.^2^ Colonoscopy is treated as the gold standard of CRC screening. However, certain limitations, such as the rate of missed lesions and incomplete coverage, objectively exist.

Transfer RNA (tRNA), as an important regulatory noncoding RNA, can generate a variety of tRNA‐derived fragments (tRFs). Available evidences indicated that tRFs were involved in the development of various diseases, including CRC.^3^, ^4^ In addition, tRFs could serve as innovative biomarkers for disease screening.^4^ Therefore, we conducted this three‐stage sequential clinical study aiming to discover potential innovative tRF biomarkers for CRC screening. Considering that approximately 85% of CRCs may be transformed from adenomas, especially from advanced adenomas (AAs),^5^ we detected tRFs in both AA and CRC. The detailed information regarding the study design and participant recruitment is shown in Supporting Information and Table S1.

Firstly, a cross‐sectional study was conducted, in which five pairs of age‐ and gender‐matched AA and CRC patients were enrolled. High‐throughput sequencing was employed to detect levels of tRFs in tissues and then to establish the pool of potential tRFs. Nine tRF subtypes were identified, among which tRF‐1, tRF‐3a, and tRF‐5c accounted for majority (Figure S1A), and the length mainly included two peaks (14–18 nt and 28–34 nt) (Figure S1B). The origin analysis indicated that tRFs mainly came from Glu‐tRNA, Gly‐tRNA, and Lys‐tRNA (Figure S1C). Differentially expressed tRFs were obtained from the comparisons of AA versus normal (332) and CRC versus normal (40) (Figure 1A). The overlapping 12 tRFs were filtered out with Venn diagram in the AA and CRC groups compared with the normal group (Figure 1B and Table S2). We chose the single upregulated tRF and five downregulated tRFs with the most significant difference for next‐stage verification by real‐time polymerase chain reaction (RT‐PCR) (Table S3).

**FIGURE 1:**
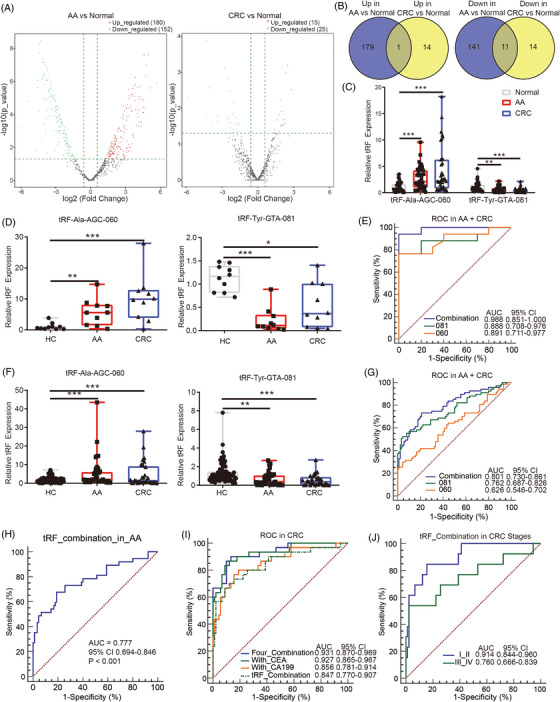
Transfer RNA (tRNA)‐derived fragments (tRFs) were promising biomarkers for screening of colorectal cancer (CRC). (A) Significantly different tRFs in pairwise comparisons were showed. (B) Overlapping tRFs were showed between the advanced adenoma (AA) group compared with the normal group and the CRC group compared with the normal group. (C) Levels of tRF‐Tyr‐GTA‐081 and tRF‐Ala‐AGC‐060 in tissues were verified by real‐time polymerase chain reaction (RT‐PCR) (normal = 40, AA = 40, and CRC = 40). (D) Levels of tRF‐Ala‐AGC‐060 and tRF‐Tyr‐GTA‐081 in the blood samples was performed by RT‐PCR (healthy control [HC] = 10, AA = 10, and CRC = 10). (E) Receiver operating characteristic (ROC) analysis of tRF‐Ala‐AGC‐060 (060), tRF‐Tyr‐GTA‐081 (081), and combination of tRF‐Ala‐AGC‐060 and tRF‐Tyr‐GTA‐081 (combination) was performed. (F) Levels of tRF‐Ala‐AGC‐060 and tRF‐Tyr‐GTA‐081 in the blood samples were verified by RT‐PCR (HC = 91, AA = 37, and CRC = 30). (G) ROC analysis of tRF‐Ala‐AGC‐060 (060), tRF‐Tyr‐GTA‐081 (081), and combination of tRF‐Ala‐AGC‐060 and tRF‐Tyr‐GTA‐081 (combination) were showed (HC = 91, AA = 37, and CRC = 30). (H) ROC analysis of combination of tRF‐Ala‐AGC‐060 and tRF‐Tyr‐GTA‐081 (tRFs combination) in AA was showed. (I) ROC analysis of tRFs combination with carcinoembryonic antigen (CEA) or carbohydrate antigen 19‐9 (CA19‐9) were performed. (J) ROC analysis of tRFs combination in early and late stages of CRC. Data are presented as the means ± standard error of means. ^*^ *p* < 0.05, ^**^ *p* < 0.01, ^***^ *p* < 0.001.

Afterwards, an enlarged cohort including 40 pairs of AA and CRC patients was established by RT‐PCR. The results indicated that tRF‐Tyr‐GTA‐081 was significantly downregulated and tRF‐Ala‐AGC‐060 was significantly upregulated in the AA and CRC groups, while others did not show any difference (Figures 1C and S1D).

Eventually, we designed a third cohort including 37 AA patients, 30 CRC patients, and 91 healthy controls (HCs) to testify above results by RT‐PCR in blood samples. Then, two steps were arranged. To be specific, an exploratory dataset consisting of 10 AA patients, 10 CRC patients, and 10 HCs was initially set to preliminarily assess the diagnostic performance, and then a validation dataset with an enlarged sample size (all participants in the third cohort) was set to further demonstrate the results. In the exploratory dataset, similar to tissue samples, tRF‐Tyr‐GTA‐081 was downregulated, while tRF‐Ala‐AGC‐060 was upregulated (Figure 1D). And the area under the curve (AUC) values of tRF‐Tyr‐GTA‐081 and tRF‐Ala‐AGC‐060 were 0.888 (95% confidence interval [CI] 0.708–0.976) and 0.891 (95% CI 0.711–0.977) in AA and CRC, respectively. Moreover, the combination also showed better diagnostic performance with an AUC of 0.988 (95% CI 0.851–1.000) (Figure 1E). Next, in the validation dataset, the levels of tRF‐Tyr‐GTA‐081 and tRF‐Ala‐AGC‐060 maintained the same trend as in the exploratory dataset (Figure 1F). And tRF‐Tyr‐GTA‐081 and tRF‐Ala‐AGC‐060 generated AUC values of 0.762 (95% CI 0.687–0.826) and 0.626 (95% CI 0.546–0.702), respectively. Similarly, the combination could further improve the diagnostic performance and achieve an AUC of 0.801 (95% CI 0.730–0.861) with sensitivity of 73.13% and specificity of 78.89% (Figure 1G). In addition, to evaluate whether tRF‐Tyr‐GTA‐081 and tRF‐Ala‐AGC‐060 in blood samples presented comparable trajectories in tissues, the correlation analysis between tumor tissues and blood samples was performed. We involved 30 pairs of AA and CRC patients with both tissue and blood samples reserved. Pearson's correlation analysis demonstrated that the tissue samples were roughly correlated with the blood samples in tRF‐Ala‐AGC‐060 level, while no significant correlation was found in tRF‐Tyr‐GTA‐081 level (Table S4). Multinomial logistic regression indicated potential association between blood tRF levels and participants’ pathological types (Table S5). These results indicated that blood tests were as feasible as histological tests.

Thereafter, the diagnostic performance of tRFs combination in AA or CRC was analyzed. In AA, the tRFs combination generated an AUC value of 0.777 with sensitivity of 67.57% and specificity of 81.11% (Figure 1H). And in CRC, the tRFs combination generated an AUC value of 0.847 with sensitivity of 73.33% and specificity of 84.44% (Figure 1I). Besides, the diagnostic performance of two conventional serum biomarkers (CEA and CA19‐9) was also investigated. CEA and CA19‐9 generated AUC values of 0.763 (sensitivity of 66.67% and specificity of 79.12%) and 0.656 (sensitivity of 60.00% and specificity of 68.13%) in CRC, respectively (Figure S2A). Additionally, we also evaluated whether the diagnostic performance would be further enhanced when assessing tRFs combination together with conventional biomarkers (CEA and CA19‐9) in CRC. The combination of tRFs with CA19‐9 could generate an AUC value of 0.856 (95% CI 0.781–0.914) with sensitivity of 80.00% and specificity of 81.11% in differentiating CRC from HC. The combination of tRFs with CEA could achieve an AUC value of 0.927 (95% CI 0.865–0.967) with sensitivity of 86.67% and specificity of 87.78%. However, the four combinations only slightly increased the diagnostic performance, with an AUC value of 0.931 (95% CI 0.870–0.969) with sensitivity of 90.00% and specificity of 86.67% (Figure 1I). These results indicated that the combination of tRF‐Tyr‐GTA‐081 and tRF‐Ala‐AGC‐060 with CEA may be served as a reliable biomarker scheme for CRC screening. Furthermore, we analyzed diagnostic performance of the tRFs combination for early‐stage CRC. Receiver operating characteristic (ROC) curve analysis showed that the tRFs combination generated AUC values of 0.914 (sensitivity of 88.24% and specificity of 84.44%) and 0.760 (sensitivity of 53.85% and specificity of 97.78%) for early and late stages of CRC, respectively (Figure 1J), which indicated that the tRFs combination could be further employed for screening of early‐stage CRC. Previous studies showed that tRFs (5′‐tRF‐GlyGCC, tRF‐Phe‐GAA‐031, and tRF‐VAL‐TCA‐002) could serve as novel biomarkers for CRC diagnosis, and 5′‐tRF‐GlyGCC showed better diagnostic performance with CEA and CA199.^3^, ^4^ In addition, 5′‐tRF‐GlyGCC was increased with the progression and metastasis of CRC.^4^ In this study, our results indicated that tRFs combination presented promising performance for CRC screening, and the diagnostic performance of tRFs combination with CEA would be further enhanced, consistent with previous studies. But we also found that the tRFs combination had better efficacy in screening for early‐stage CRC than late‐stage CRC.

To explore the potential regulatory mechanism, we identified probable targeted mRNAs and built tRF‐mRNA networks (Figure S2B). The potential regulatory pathways mainly included the PI3K‐Akt signaling pathway and Ras signaling pathway (Figure S2C).

In conclusion, we found an innovative tRFs combination (tRF‐Tyr‐GTA‐081 and tRF‐Ala‐AGC‐060) with satisfactory diagnostic performance and sensitivity in CRC. The performance of tRFs combination would be further enhanced when evaluated together with CEA. Moreover, tRFs combination presented promising performance for early‐stage CRC screening as well. Therefore, this scheme would provide a new choice for early‐stage CRC screening and benefit CRC management.

## AUTHOR CONTRIBUTIONS

G.J., Y.X., and Y.D. conceived, designed, and supervised the study. Y.D., L.D., and Y.X. collected samples. Y.D., L.D., W.Z., and J.X. performed the experiments and analyzed the data. Y.D., W.Z., and G.J. wrote the paper and edited and revised the paper. All authors reviewed and approved the final manuscript.

## CONFLICT OF INTEREST STATEMENT

The authors declare no conflicts of interest.

## FUNDING INFORMATION

Shanghai Rising‐Star Program (21QA1409000); Shanghai Frontier Research Base of Disease and Syndrome Biology of Inflammatory cancer transformation (2021KJ03‐12).

## ETHICS STATEMENT

This study was approved by the Ethics Committee of Longhua Hospital (2019LCSY020), and informed consent was obtained from all participants.

## Supporting information

Supporting Information

## Data Availability

All data generated or analyzed during this study are included in this published article and its Supporting Information.
