# Supplementary material for: tRNA‐derived fragments are promising biomarkers for screening of early colorectal cancer
Source: MedComm (2020). 2023 May 3;4(3):e227. doi: 10.1002/mco2.227 (PMC10156994; doi:10.1002/mco2.227)
Supplement: Supplementary file 1 — Supporting Information [file MCO2-4-e227-s001.docx]

tRNA-derived fragments are promising biomarkers for screening of early colorectal cancer

Yanqi Dang^1#*^, Liang Dai^1,3#^, Jingjuan Xu^1^, Wenjun Zhou^1^, Yangxian Xu^2*^, Guang Ji^1*^

^1^ Institute of Digestive Diseases, Longhua Hospital, China-Canada Center of Research for Digestive Diseases (ccCRDD), Shanghai University of Traditional Chinese Medicine, Shanghai, 200032, China.

^2^ Department of General Surgery, Longhua Hospital, Shanghai University of Traditional Chinese Medicine, Shanghai 200032, China

^3^ Clinical Research Academy, Peking University Shenzhen Hospital, Peking University, Shenzhen, 518036, China

Yanqi Dang and Liang Dai contributed equally to this work.

Correspondence to: Yangxian Xu, email: xuyangxian@163.com;

Yanqi Dang, email: [dangyanqi9022@126.com](mailto:dangyanqi9022@126.com);

Guang Ji, email: [jiliver@vip.sina.com](mailto:jiliver@vip.sina.com); [jg@shutcm.edu.cn](mailto:jg@shutcm.edu.cn)

**Materials and Methods**

**Study design and sample collection**

All AA and CRC participants were recruited from departments of endoscopy and gastrointestinal surgery from May 2019 to December 2019. The diagnostic criteria of AA were defined as adenoma diameter > 10mm, with or without villous texture or high-grade neoplasia. The diagnosis of CRC should be confirmed based on the pathological evidences. The stage of CRC was determined based on the eighth edition American Joint Committee on Cancer (AJCC) CRC staging system. Early CRC was defined as CRCs in stages I and II, while late CRC was CRCs in stages III and IV. Participants combined with other primary cancers were excluded. A total of 47 AA patients and 40 CRC patients were included. The study also recruited healthy volunteers to serve as healthy controls (HCs). 91 healthy controls met the following criteria and were included: (1) normal ranges of routine laboratory tests (including blood routine test, liver and kidney function and fasting blood glucose), (2) absence of any colonoscopy evidences of adenoma and neoplasm. The study was approved by the Ethics Committee of Longhua Hospital (2019LCSY020), and the informed consent was obtained from all participants.

For AA and CRC patients, samples of AA, CRC and normal distant tissues (at least 3cm from the tumor margin) were obtained from endoscopic polypectomy and surgery, snap-frozen in liquid nitrogen and stored at -80 °C before detection. Blood samples were obtained based on voluntariness principle. Based on the matching principle of age and gender, firstly, we conducted a cross-sectional study of 5 pairs of AA and CRC patients, to find out the potential tRFs and tiRNAs. Afterwards, an enlarged cohort including 40 pairs of AA and CRC patients was used to verify the results. Blood and serum samples were available in 37 AA patients and 30 CRC patients. Hence, a third cohort including 37 AA patients, 30 CRC patients and 91 HCs was established to testify above results.

**RNA sequencing and data analysis**

Total RNA was extracted from five normal distant tissues, AA and CRC tissues using TRIzol reagent (Life Technologies, CA, USA). The RNA quality was assessed using Nanodrop^TM^ instrument. The RNA sequencing data were acquired using Illumina Hiseq 4000. Clean reads were obtained and aligned to Human Genome. The differentially expressed tRFs were yielded using DEseq2 software with fold change more than 2 and *P* value less than 0.05. The overlapping differentially expressed tRFs between the AA and CRC groups compared with the normal group were obtained by Venn diagram, which were used for further analysis. Networks of tRF-Tyr-GTA-081 and tRF-Ala-AGC-060 were analyzed by Cytoscape software. Kyoto Encyclopedia of Genes and Genomes (KEGG) analyses was performed on the differentially expressed tRF-Tyr-GTA-081 and tRF-Ala-AGC-060-associated genes.

**Validation of candidate tRFs using real-time polymerase chain reaction (RT-PCR)**

Total RNA of tissue and blood samples was extracted using TRIzol reagent or TRIzol LS reagent (Life Technologies, CA, USA) following the manufacturer’s instruction. Total RNA was treated to remove some RNA modifications using rtStar™ tRF&tiRNA Pretreatment Kit (Cat# AS-FS-005, Arraystar) following the manufacturer’s instruction and previous studies ^1-3^, and then reverse-transcribed in 50 °C for one hour using rtStar™ First-Strand cDNA Synthesis Kit (3’ and 5’ adaptor) (Cat# AS-FS-003, Arraystar). Six differentially expressed tRFs were examined by RT-PCR followed 95 °C for 10 s, 60 °C for 10 s, and 72 °C for 10 s, for 40 cycles using SYBR Green PCR Master Mix (Applied Biosystems, Foster City, CA, USA). Expression of tRFs was normalized to U6, and data were analyzed using the 2^−ΔΔCt^ method. Primers sequences of tRFs were listed in Table S3.

**Statistical analysis**

Data were presented as mean ± SEM and analyzed using a one-way analysis of variance (ANOVA) in SPSS Statistics v20.0. The correlation analysis of the tRFs levels in the tumor tissues and blood samples was conducted by Pearson’s correlation analysis. Multinomial logistic regression was applied to evaluate the association between the selected tRFs and pathological types. *P* value less than 0.05 was considered statistically significant. The receiving operational curve (ROC) was generated and area under curve (AUC) was calculated to assess the diagnostic performance of tRFs with MedCalc 18.11 software.

Table S1 Demographic characteristics of included participants

|  | AA (n=47) | CRC (n=40) | HC (n=91) |
| --- | --- | --- | --- |
| Age, mean (sd), yrs | 60.74 (8.92) | 63.48 (12.79) | 57.77 (15.42) |
| Female, n(%) | 20 (42.55%) | 13 (32.50%) | 47 (51.65%) |
| Male, n(%) | 27 (57.45%) | 27 (67.50%) | 44 (48.35%) |
| Localization n (%) |  |  |  |
| Right (cecum, ascending, transverse) | 12 (25.53%) | 10 (25%) | - |
| Left (descending, sigmoid) | 32 (68.09%) | 14 (35%) | - |
| Rectum | 3 (6.38) | 16 (40%) | - |
| TNM stage, n (%) |  |  |  |
| I | - | 11 (27.5%) | - |
| II | - | 8 (20%) | - |
| III | - | 16 (40%) | - |
| IV | - | 5 (12.5%) | - |

Table S2. Overlapping tRFs between the AA group compared to the normal group and the CRC group compared to the normal group.

| **RNAs** | **AA vs Normal** | | | **CRC vs Normal** | | |
| --- | --- | --- | --- | --- | --- | --- |
|  | **Fold change** | ***P* value** | **Regulation** | **Fold change** | ***P* value** | **Regulation** |
| tRF-Ala-AGC-060 | 8.809027766 | 0.000129522 | Up | 3.909324985 | 0.031371638 | Up |
| tRF-Arg-TCG-015 | 0.016206641 | 0.000642721 | Down | 0.018273066 | 0.005322515 | Down |
| tRF-Arg-TCG-042 | 0.040208845 | 0.016099234 | Down | 0.0516043 | 0.01810768 | Down |
| tRF-Ser-CGA-001 | 0.118264806 | 0.000999995 | Down | 0.053418953 | 0.000130186 | Down |
| tRF-Tyr-GTA-081 | 0.074508065 | 0.021210374 | Down | 0.097699717 | 0.024429785 | Down |
| tRF-Cys-GCA-027 | 0.121727225 | 0.015295976 | Down | 0.074118728 | 0.029266619 | Down |
| tRF-Arg-TCG-045 | 0.054296368 | 0.004136002 | Down | 0.13456493 | 0.043035462 | Down |
| tRF-Arg-TCG-004 | 0.20128607 | 0.030661691 | Down | 0.188593953 | 0.024517559 | Down |
| tRF-Gly-CCC-020 | 0.073455755 | 1.28916E-05 | Down | 0.200792783 | 0.003394145 | Down |
| tiRNA-Leu-TAA-001 | 0.07195075 | 0.000168144 | Down | 0.229033175 | 0.014541273 | Down |
| tRF-Ser-AGA-004 | 0.250436329 | 0.004436765 | Down | 0.247658199 | 0.00688903 | Down |
| tRF-Gly-CCC-021 | 0.056753443 | 7.3957E-07 | Down | 0.344039483 | 0.027489057 | Down |

Table S3. Primers sequences of tRFs in real-time PCR experiments.

| RNAs | Primer(5'->3') |
| --- | --- |
| tRF-Ala-AGC-060 | Forward: GTCCGACGATCTCCCCAGTA  Reverse: TGTGCTCTTCCGATCTTGGT |
| tRF-Arg-TCG-015 | Forward: AGTTCTACAGTCCGACGATCGA  Reverse: CTCTTCCGATCTAATGAGTCGAAA |
| tRF-Arg-TCG-042 | Forward: AGAGTTCTACAGTCCGACGATCAT  Reverse: CTCTTCCGATCTAATGAGTCGAAA |
| tRF-Ser-CGA-001 | Forward: GTCCGACGATCGTCACGGT  Reverse: TGCTCTTCCGATCTACTCGGC |
| tRF-Tyr-GTA-081 | Forward: GAGTTCTACAGTCCGACGATCT  Reverse: CTCTTCCGATCTAGATTTACAGTC |
| tRF-Cys-GCA-027 | Forward: AGTTCTACAGTCCGACGATCCT  Reverse: TGCTCTTCCGATCTAAAAAAGGC |
| U6 | Forward: GCTTCGGCAGCACATATACTAAAAT  Reverse: CGCTTCACGAATTTGCGTGTCAT |

Table S4. Correlation analysis between tumor tissues and blood samples in tRF-Tyr-GTA-081 and tRF-Ala-AGC-060 levels

|  | **Correlation coefficient** | ***P* value** |
| --- | --- | --- |
| tRF-Tyr-GTA-081 | -0.232 | 0.074 |
| tRF-Ala-AGC-060 | 0.262 | 0.043 |

Table S5. Association between blood tRFs levels and participants’ pathological types

|  |  | **OR** | **95% CI** | ***P* value** |
| --- | --- | --- | --- | --- |
| tRF-Tyr-GTA-081 | Normal | Ref | / | / |
|  | AA | 0.44 | 0.226-0.866 | 0.017 |
|  | CRC | 0.33 | 0.141-0.748 | 0.008 |
| tRF-Ala-AGC-060 | Normal | Ref | / | / |
|  | AA | 1.15 | 0.995-1.316 | 0.058 |
|  | CRC | 1.13 | 0.980-1.302 | 0.092 |


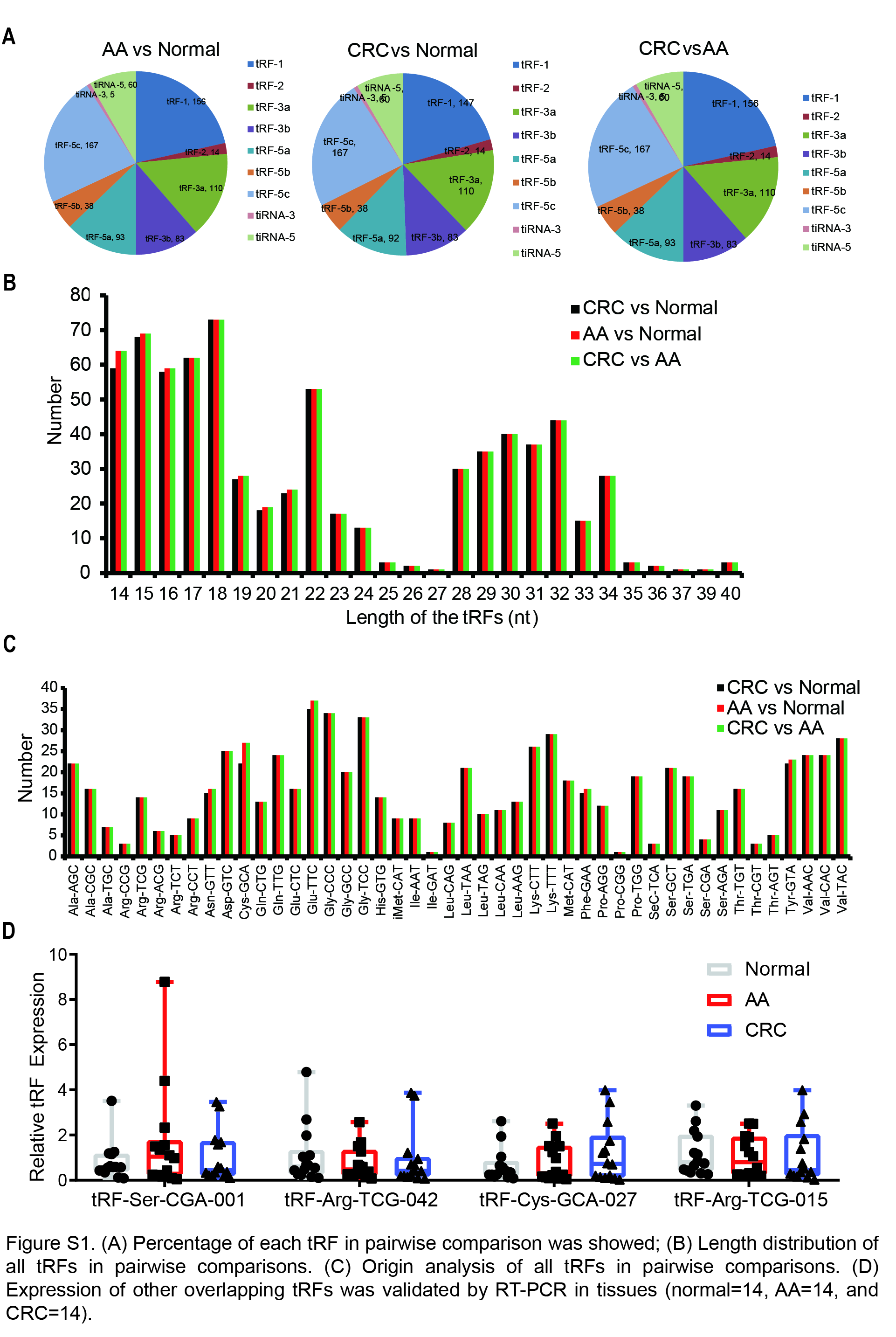


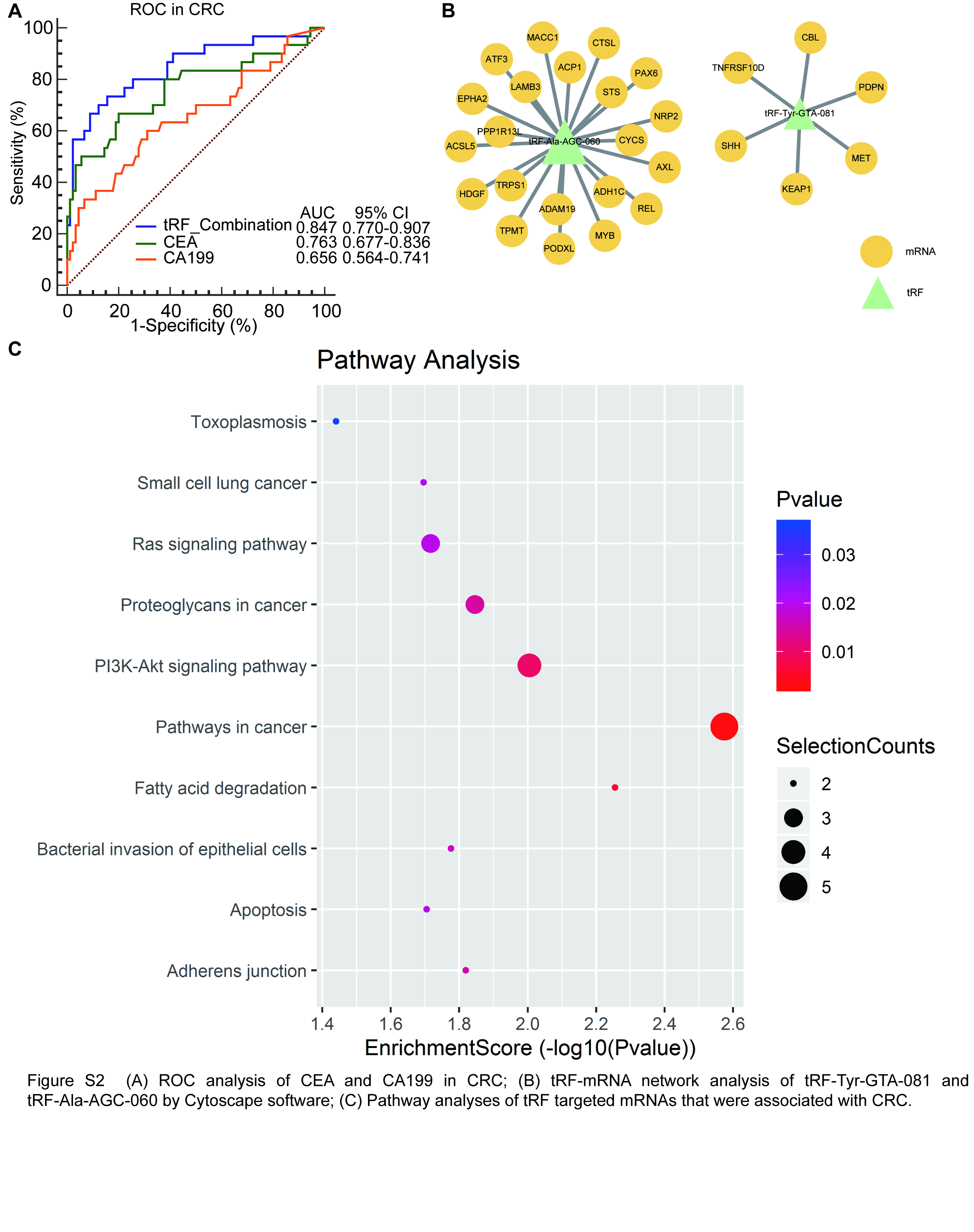


Figure S1. (A) Percentage of each tRF in pairwise comparison was showed; (B) Length distribution of all tRFs in pairwise comparisons. (C) Origin analysis of all tRFs in pairwise comparisons. (D) Expression of other overlapping tRFs was validated by RT-PCR in tissues (normal=14, AA=14, and CRC=14).

Figure S2 (A) ROC analysis of CEA and CA199 in CRC; (B) tRF-mRNA network analysis of tRF-Tyr-GTA-081 and tRF-Ala-AGC-060 by Cytoscape software; (C) Pathway analyses of tRF targeted mRNAs that were associated with CRC.

References:

1. Mo D, He F, Zheng J, Chen H, Tang L, Yan F. tRNA-Derived Fragment tRF-17-79MP9PP Attenuates Cell Invasion and Migration via THBS1/TGF-beta1/Smad3 Axis in Breast Cancer. *Front Oncol.* 2021;11:656078.

2. Han Y, Peng Y, Liu S, et al. tRF3008A suppresses the progression and metastasis of colorectal cancer by destabilizing FOXK1 in an AGO-dependent manner. *J Exp Clin Cancer Res.* 2022;41(1):32.

3. Lu S, Wei X, Tao L, et al. A novel tRNA-derived fragment tRF-3022b modulates cell apoptosis and M2 macrophage polarization via binding to cytokines in colorectal cancer. *J Hematol Oncol.* 2022;15(1):176.
